# Effects of social context information on neural face processing in youth with social anxiety disorder

**DOI:** 10.1111/jcpp.70026

**Published:** 2025-08-04

**Authors:** Anna‐Lina Rauschenbach, Vera Hauffe, Jakob Fink‐Lamotte, Brunna Tuschen‐Caffier, Julian Schmitz

**Affiliations:** ^1^ Department of Clinical Child and Adolescent Psychology Leipzig University Leipzig Germany; ^2^ Department of Clinical Psychology and Psychotherapy University of Freiburg Freiburg im Breisgau Germany; ^3^ Department of Clinical Psychology University of Potsdam Potsdam Germany

**Keywords:** children, social anxiety, face processing, context effects, event‐related potentials

## Abstract

**Background:**

Social anxiety disorder (SAD) in youth is associated with significant psychosocial impairments; however, the cognitive and neural mechanisms that maintain it, particularly during childhood and adolescence, remain underexplored. Cognitive models emphasize the role of altered face processing, and neutral facial expressions may be perceived as threatening. Due to their ambiguous nature, contextual cues may play a particularly important role in interpretation.

**Methods:**

We presented neutral child faces paired with social context information varying in valence (negative, neutral, positive) while continuous EEG was recorded. Subjective valence ratings and neural responses (P100, N170, and LPP) were assessed in children and adolescents aged 10–15 years with SAD (*n* = 53), clinical controls with specific phobias (SP; *n* = 41), and healthy controls (HC; *n* = 61).

**Results:**

Overall, context information affected both the subjective and neural responses to neutral faces in all children and adolescents, for example, more negative ratings for negatively contextualized faces. Further, participants with SAD generally rated all faces as more negative compared to HCs. Neurally, they showed lower N170 amplitudes compared to both control groups in response to all neutral faces, independent of the context valence. However, only younger children (aged 10–12 years) with SAD showed higher LPP amplitudes than younger HCs.

**Conclusions:**

Processing biases seem to be already present in children and adolescents with SAD, both at the subjective and neural level. Social context information influences neutral face processing but is independent of psychopathology. Future studies examining age effects are needed to investigate whether childhood reflects a particularly sensitive period for the development of processing biases.

Social anxiety disorder (SAD) is one of the most prevalent mental disorders among youth, often resulting in significant adverse psychosocial outcomes and comorbidities (Beesdo‐Baum & Knappe, 2012; Szafranski, Talkovsky, Farris, & Norton, 2014). Yet, relatively little is known about the cognitive and neural mechanisms that maintain it during childhood and adolescence (Halldorsson & Creswell, 2017). For adults, cognitive models suggest that the fear of (negative) evaluations plays a key role in the maintenance of SAD (Clark & Wells, 1995; Rapee & Heimberg, 1997). In social interactions, facial expressions frequently convey critical information regarding social evaluations, and theoretical models and empirical evidence suggest that socially anxious individuals show processing biases when confronted with potentially threatening faces, including facilitated attention allocation to threat‐related cues and distortions in interpretation (Clark & Wells, 1995; Heinrichs & Hofmann, 2001).

In this field, the investigation of event‐related potentials (ERPs) via EEG can provide valuable insights into early automatic electrocortical responses to facial expressions. The frequently investigated P100 reflects an occipital‐positive deflection associated with early attention allocation (Luck, 2005). The occipito‐temporal N170 is also of interest, as it represents a negative face‐specific potential involved in the structural encoding of faces (Bentin, Allison, Puce, Perez, & McCarthy, 1996; Rossion & Jacques, 2008). Additionally, the occipito‐parietal late positive potential (LPP) reflects more controlled processes, such as sustained motivated attention (Cuthbert, Schupp, Bradley, Birbaumer, & Lang, 2000; Schupp et al., 2000).

Face processing studies in adults with SAD have yielded heterogeneous results. While most studies reported an association between social anxiety and higher P100 amplitudes, results regarding the N170 are conflicting, and few studies analysed LPPs, with a trend towards higher LPP amplitudes in socially anxious individuals (e.g., Harrewijn, Schmidt, Westenberg, Tang, & Van Der Molen, 2017). Due to important brain maturation processes and general age effects observed in ERPs (Luna, Garver, Urban, Lazar, & Sweeney, 2004; MacNamara et al., 2016), these findings may not generalize to younger populations, and relatively few studies have examined neural face processing in childhood SAD. Moreover, those studies also present inconsistent findings: While some found no difference between children with SAD and controls regarding the P100 and N170 (Keil, Uusberg, Blechert, Tuschen‐Caffier, & Schmitz, 2018; Schwab & Schienle, 2017a), others reported lower N170 amplitudes in youth with SAD compared to healthy controls (HCs; Rauschenbach, Hauffe, Fink‐Lamotte, Tuschen‐Caffier, & Schmitz, 2024) and in socially anxious compared to non‐socially anxious children (Wauthia, Rossignol, Blekic, Lefebvre, & D'Hondt, 2023). Regarding later processing stages, two studies found higher LPPs in children with SAD compared to HCs (Kujawa, MacNamara, Fitzgerald, Monk, & Phan, 2015; Schwab & Schienle, 2017a). However, this association only reached trend level in a subsample of younger children (Rauschenbach et al., 2024) and was not found in another study (Keil et al., 2018).

These inconsistencies may stem from methodological limitations, including small samples, a small number of trials, and wide age ranges. In addition, the frequent lack of clinical controls precludes conclusions about transdiagnostic effects. Lastly, child faces are associated with peer relationships and peer evaluation, which become increasingly important during adolescence (Oerter & Montada, 2002), but in most paradigms, adult or schematic faces were presented.

Beyond these limitations, the aforementioned studies all examined faces in isolation. However, emotional faces in everyday interactions are typically situated within complex contexts, and contextual cues are particularly relevant when processing ambiguous expressions, such as neutral faces (Schwarz, Wieser, Gerdes, Mühlberger, & Pauli, 2013; Wieser et al., 2014). At the same time, social anxiety is associated with biases resulting in more negative interpretations of neutral faces (Peschard & Philippot, 2017; Yoon & Zinbarg, 2008); thus, it is crucial to understand the interplay between social anxiety and the reliance on contextual cues when processing neutral faces.

In Wieser and Moscovitch's (2015) EEG study, participants with high levels of social anxiety showed higher P100 and lower N170 amplitudes in response to neutral faces paired with negative, neutral, and positive context information. Additionally, they had higher LPPs for negatively contextualized faces, while participants with low social anxiety had enhanced LPPs for positively contextualized faces. In contrast, when angry child faces were embedded in situational contexts (Schwab & Schienle, 2017b) or paired with a reappraisal task (Keil, Tuschen‐Caffier, & Schmitz, 2022), children with SAD did not differ from controls in their early neural response (P100 and N170). Yet, they showed higher parietal LPPs compared to HCs independent of the context (Schwab & Schienle, 2017b), while in the other study, LPPs did not differ between children with SAD, clinical controls with mixed anxiety disorders, and HCs (Keil et al., 2022). These discrepancies regarding the modulation of the LPP may be due to the presentation of angry faces in both paediatric studies, which are less ambiguous, may generally be perceived as threatening, and may therefore not necessitate the reliance on context information.

The present study is the first to examine the subjective and neural response to children's neutral faces and their modulation through contextual information in a large sample of children and adolescents with SAD, compared to HCs and a homogenous clinical control group with specific phobias (SP). SPs are characterized by circumscribed, intense fears of specific objects, animals or insects, or situations, thus exceeding normative developmental fears due to their persistence and clinical significance (American Psychiatric Association, 2013). Although youth with SP generally have lower levels of psychopathology than youth with SAD, this group represents a clinically valuable control group. Their inclusion allows the investigation of differences in neural reactivity to socially relevant stimuli not only in comparison with HCs but also in comparison to another anxiety disorder. Therefore, a clinical control group with SP could shed light on the question of whether subjective and neural processing biases are already evident at lower levels of clinical anxiety and psychopathology.

Behaviourally, we hypothesized that youth with SAD would rate negatively contextualized faces as more negative compared to both control groups (Wieser & Moscovitch, 2015). Neurally, we expected youth with SAD to show reduced early attentional processing of neutral faces compared to both control groups, which would be mainly evident in lower N170 amplitudes independent of the context valence (Rauschenbach et al., 2024; Wieser & Moscovitch, 2015). We further assumed that youth with SAD would show higher LPPs compared to both control groups in response to negatively contextualized faces, as they are the most threatening (Wieser & Moscovitch, 2015). Lastly, we examined age as a potential moderator.

## Methods

### Participants

We recruited participants in two German cities via local schools and the cities' register of residents for a two‐centre project on childhood SAD. The final sample consisted of 155 children and adolescents (79 female) aged 10–15 years. An additional 11 children were excluded from final analyses due to incomplete EEG experiments (*n* = 7), irregularities in the EEG (*n* = 1), or exclusion criteria (*n* = 3). The study was approved by the local ethics committee, and all youths and their parents were informed about the three‐session project [session 1: diagnostic interview; session 2: eyetracking (Hauffe, Rauschenbach, Fassot, Schmitz, & Tuschen‐Caffier, 2025); session 3: another EEG experiment (Rauschenbach et al., 2024) followed by this experiment]. All parents provided informed consent, and assent was obtained from participating youth. Children and adolescents received age‐appropriate vouchers for their participation worth €70, while parents were paid €30.

The inclusion criterion was a primary diagnosis of SAD (SAD group; *n* = 53), a primary SP diagnosis without comorbid mood or anxiety disorders (SP group; *n* = 41) or no lifetime mental disorder (HCs; *n* = 61). Exclusion criteria for all groups included psychotic episodes, autism spectrum disorder, severe depressive episodes, suicidality, current or past psychotherapeutic treatment, psychotropic drug intake, pervasive developmental or neurological disorders, severe visual impairment and an IQ below 80 (assessed with the short version of the Culture Fair Intelligence Test, CFT 20‐R; Weiß, 2006). Parents first completed a brief phone screening. To further assess the diagnostic status according to DSM‐5 (American Psychiatric Association, 2013), eligible youth and their parents completed online questionnaires and were interviewed separately using the Structured Diagnostic Interview for Mental Disorders in Children (Schneider, Pflug, In‐Albon, & Margraf, 2017), which has shown excellent interrater reliability for lifetime anxiety disorders (Neuschwander, In‐Albon, Adornetto, Roth, & Schneider, 2013). Trained graduate or doctoral students conducted the interviews, which were videotaped and supervised by licensed clinical psychologists.

### Psychometric measures

Participants filled out the German version of the revised Social Anxiety Scale for Children (SASC‐R‐D; Melfsen & Florin, 1997), which assesses symptoms related to fear of negative evaluation and social avoidance and distress. Phobic fears were measured with the German version of the revised Fear Survey Schedule for Children (FSSC‐R; Döpfner, Schnabel, Goletz, & Ollendick, 2006). Lastly, youth completed the German version of the Children's Depression Inventory (CDI; Stiensmeier‐Pelster, Braune‐Krickau, Schürmann, & Duda, 2014), focusing on depressive symptoms and their severity. Parents reported on their children's psychopathology based on the Child Behavior Checklist (CBCL; Döpfner, Plück, & Kinnen, 2014). In the present sample, internal consistency for psychometric measures was good to excellent (*α* = .83–.96).

### Stimulus material and procedure

Overall, 36 photographs of neutral child faces (50% female) with direct gaze were selected from the National Institute of Mental Health Child Emotional Faces Picture Set (Egger et al., 2011) and converted to gray‐scale. The paradigm was based on the study by Wieser and Moscovitch (2015). The respective contextual sentences were adapted to be more child friendly, and 36 sentences with the same grammatical structure, varying in valence (negative, neutral and positive) and gender (female or male pronoun) were selected based on their valence ratings in a pilot study (*n* = 28, aged 10–15 years). As in the original study (Wieser & Moscovitch, 2015), this resulted in six sentences per context valence and gender (Table 1). Each individual face was paired with one context category (negative, neutral and positive) and presented once with each of the according context sentences. Consequently, each individual face was shown six times throughout the experiment. In addition, the gender of the stimuli was balanced so that six female and six male faces were assigned to each context valence, and the assignment was counterbalanced across participants. This procedure resulted in 72 trials per context valence: picture (6) × sentence (6) × gender (2). The order of the stimuli was randomized with the restriction that the same context valence was not presented in more than three consecutive trials.

**Table 1:** Overview of context sentences with different valence categories

	Negative valence	Positive valence	Neutral valence
He/she…	Doesn't think you are nice	Thinks you are very nice	Thinks your shoes are black
	Thinks you can't be trusted	Thinks you can be trusted	Thinks you like holidays
	Thinks you are stupid	Thinks you are very smart	Thinks your t‐shirt is white
	Thinks you are embarrassing	Thinks you are cool	Thinks you are right‐handed
	Finds your voice unpleasant	Finds your voice pleasant	Thinks you play an instrument
	Thinks your friends are stupid	Thinks your friends are nice	Thinks you are sitting on a chair

In the study, sentences were presented in German; this list represents a translation.

**Figure 1:**
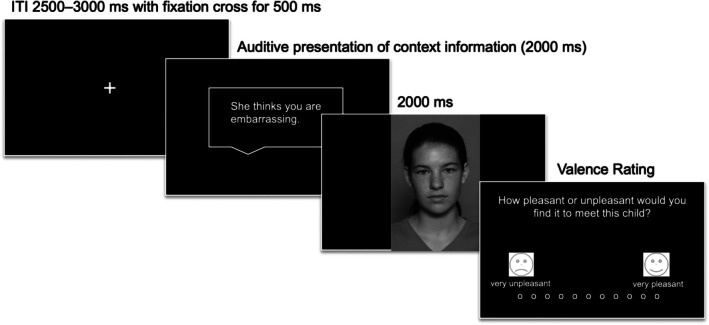
Schematic diagram of the EEG experiment. Pictures of neutral child faces were preceded by auditive contextual information (negative, neutral and positive). In one third of the trials, a valence rating followed

During the EEG recording, participants sat on a comfortable chair in a sound‐attenuated, temperature‐controlled room and were instructed to attend to the stimuli. The experiment consisted of four practice trials followed by 216 trials arranged into four blocks with breaks in between. Figure 1 shows an exemplary trial sequence. Each trial started with an inter‐trial interval (2,500–3,000 ms) in which a fixation cross was presented for 500 ms in the centre of the screen. Next, the auditive context sentence was presented for 2,000 ms and then a picture of a child with a neutral facial expression was shown for another 2,000 ms. In one third of the trials (randomized), participants had to rate the valence of the previously shown face; they were asked how pleasant or unpleasant they would find it to meet the respective child (0 = *very unpleasant* to 10 = *very pleasant*; internal consistency for valence ratings per group: *α* = .52–.82). Before and after the experiment, participants had to rate their state arousal (0 = *no anxiety* to 10 = *extreme anxiety*). The stimulus presentation was programmed in Matlab version 9.7.0 (The MathWorks Inc., 2019), using the Psychophysics Toolbox extensions (Kleiner, Brainard, & Pelli, 2007).

### EEG recording and data reduction

We recorded continuous EEG with a 64‐channel active electrode actiCAP system (Brain Products GmbH, Gilching, Germany) and BrainVision Recorder at a sampling rate of 1,000 Hz. One study centre used the BrainAmp amplifier with a reference electrode at FCz; the other set‐up included a QuickAmp amplifier with an average reference. Electrodes were placed following the 10–20 system with the ground electrode positioned at FPz. In addition, four electrooculogram electrodes recorded eye movement. We kept electrode impedances below 20 kΩ.

Offline data was pre‐processed using BrainVision Analyzer version 2.2.0 (Brain Products GmbH). First, data were down sampled to 500 Hz. After a visual inspection, we removed problematic channels and re‐referenced to an average reference. Next, we applied a band‐pass filter from 0.1 to 30 Hz. Ocular artefacts were corrected based on a semiautomatic independent component analysis (ICA), using a restricted infomax algorithm trained on 400–500 s of artefact‐free data. After the ICA, removed channels were interpolated. EEG data were time‐locked to the stimulus onset, segmented into epochs 200 ms prestimulus onset to 2000 ms poststimulus onset, and a baseline correction was applied. Artifactual epochs were identified based on a threshold criterion of ±125 μV and a maximal allowed voltage step of 50 μV/ms and then removed. This resulted in an epoch retention rate of 89% (*SD* = 10%), which did not differ among groups or context valence (*p*s > .41).

**Figure 2:**
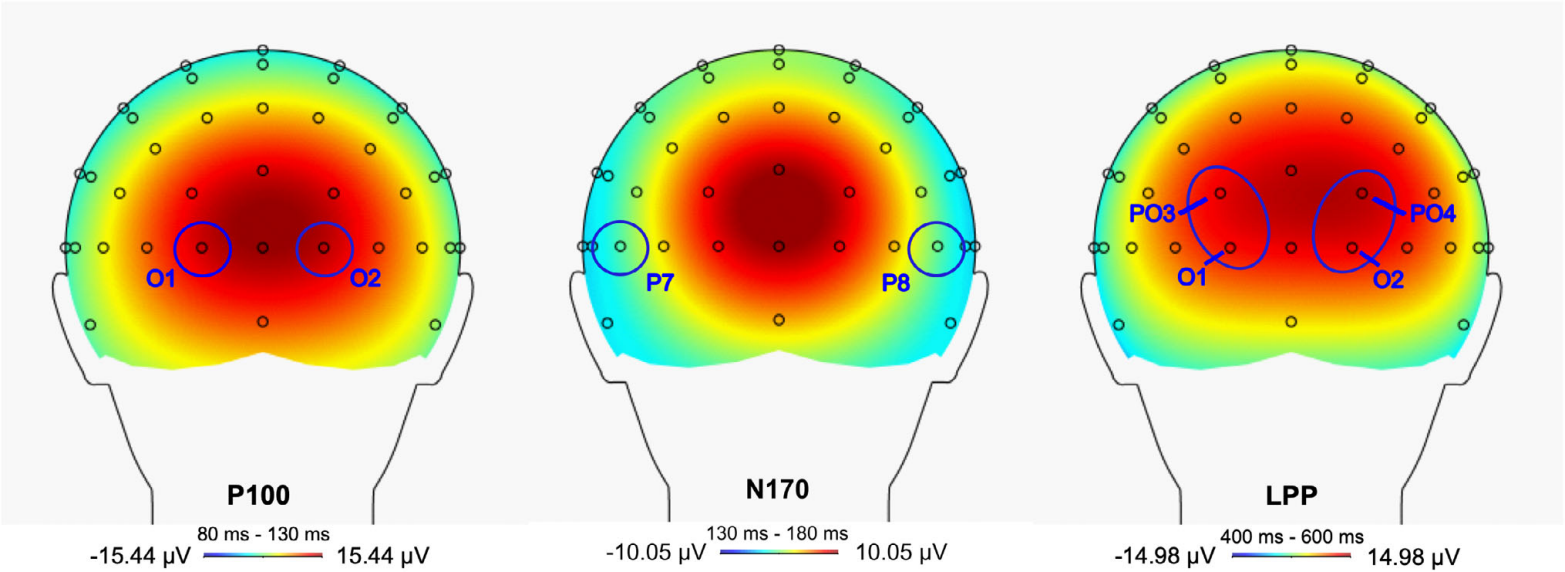
Scalp distribution of ERPs viewed from the back reflective of the response to faces averaged across all participants and context valences. The circles represent included electrodes

For the ERPs, we chose cortical positions and time windows based on previous studies (e.g., Keil et al., 2022; Kolassa & Miltner, 2006; Schmitz, Scheel, Rigon, Gross, & Blechert, 2012), in addition to visual inspections of grand averages and scalp distributions (Figure 2). We then extracted the averaged activity within the chosen time windows. We measured P100 at O1 and O2, 80–130 ms poststimulus onset. We quantified the N170 between 130 and 180 ms at P7 and P8. As previous research (Schwab & Schienle, 2017a) and the scalp distribution pointed to a potential lateralization, LPPs were analysed over lateralized occipital–parietal sites, namely O1 and PO3, as well as O2 and PO4, between 400 and 600 ms. Finally, we averaged epochs across electrodes separately for each participant and valence category. Cronbach's alpha (Thigpen, Kappenman, & Keil, 2017) and split‐half reliability estimates indicated excellent reliability for ERPs (*α* = .97–.98 and *r* = .86–.87).

### Statistical analyses

In line with previous research (e.g., Dennis & Hajcak, 2009) and to reduce model complexity, age was included in the analyses as a two‐level factor to capture potential age effects. As 13 years is the median age of onset for SAD (Kessler, Wai, Demler, & Walters, 2005), we used this age to split the sample (younger: 10–12 years, *n* = 79; older: 13–15 years, *n* = 76). A 3 (Group) × 2 (Age) × 3 (Context Valence) mixed‐design univariate analysis of variance (ANOVA) was calculated for valence ratings. We also computed 3 (Group) × 2 (Age) × 3 (Context Valence) mixed‐design ANOVAs for neural outcomes (P100, N170 and LPP). For the potentially lateralized LPP, we included the within‐factor hemisphere (left vs. right). We ran all statistical analyses in RStudio version 4.4.1 (Posit Team, 2024), applied Greenhouse–Geisser corrections for violations of sphericity, and reported corrected *p*‐values and degrees of freedom. Furthermore, to control for false discovery rates due to multiple comparisons, the Benjamini–Hochberg procedure (Benjamini & Hochberg, 1995) was applied to ANOVAs computed for ERPs, and adjusted *p*‐values are reported. Partial eta square ($\eta_{p}^{2}$) represents the effect sizes. Post hoc Tukey tests for pairwise comparisons were calculated for significant main or interaction effects, and we reported trends of *p* < .10 for post hoc analyses.

## Results

### Sample characteristics

Sample characteristics and details on comorbidities are summarized in Table 2. Overall, 68% of youth with SAD had a comorbid diagnosis, with 10 participants meeting criteria for a second comorbid disorder. As predicted, youth with SAD reported higher levels of social anxiety, depression and phobic fears compared to both control groups. Similarly, parent reports showed higher levels of general psychopathology and internalizing symptoms. Youth with SP differed from HCs regarding phobic fears, overall psychopathology and internalizing symptoms. Groups did not differ in gender, age or cognitive abilities.

**Table 2:** Sample characteristics per group

	SAD (*n* = 53)	SP (*n* = 41)	HC (*n* = 61)	Statistics	
	*n* (%)	*n* (%)	*n* (%)	*χ* ^2^ (*df* = 2)	*p*
Gender (female)	27 (50.9)	21 (51.2)	31 (50.8)	0.002 *ns*	–
Comorbid diagnoses	36 (67.9)	3 (7.3)	–		
SP	26 (49.1)	–	–		
GAS	8 (15.1)	–	–		
Separation anxiety D	2 (3.8)	–	–		
OCD	1 (1.9)	–	–		
AD(H)D	3 (5.7)	–	–		
Tic	1 (1.9)	1 (2.4)	–		
Depressive disorders	3 (5.7)	–	–		
Sleep disorder	1 (1.9)	–	–		
Elimination disorder	–	2 (4.9)	–		
ODD	1 (1.9)	–	–		

	*M* (*SD*)	*M* (*SD*)	*M* (*SD*)	*F*	Posthoc Tukey
Age	12.70 (1.68)	12.29 (1.58)	12.51 (1.66)	0.70 *ns*	–
CFT‐20‐R	108.25 (13.99)	107.44 (7.83)	106.79 (11.08)	0.23 *ns*	–
Social anxiety^a^	53.19 (10.82)	36.29 (9.06)	33.10 (7.40)	65.77***	SAD > SP/HC**
Phobic fears^b^	62.66 (23.21)	45.83 (25.67)	26.15 (18.05)	43.94***	SAD > SP > HC**
Depressive symptoms^c^	17.04 (8.05)	8.98 (5.37)	6.31 (5.00)	34.99***	SAD > SP/HC***
Psychopathology^d^	34.75 (18.46)	17.90 (11.33)	8.31 (6.10)	56.38***	SAD > SP > HC***
Internalizing	17.02 (8.70)	7.24 (5.46)	2.31 (2.37)	80.38***	SAD > SP > HC***
Externalizing	6.53 (5.28)	3.90 (3.02)	2.52 (2.88)	12.48***	SAD > HC***

Note that the German version of the questionnaires was used. AD(H)D, Attention‐Deficit/(Hyperactivity) Disorder; CFT‐20‐R, revised German Culture Fair Intelligence Test; GAS, Generalized Anxiety Disorder; HC, Healthy Control; *ns*, nonsignificant; OCD, Obsessive‐Compulsive Disorder; ODD, Oppositional Defiant Disorder; SAD, Social Anxiety Disorder; Separation anxiety ., Separation Anxiety Disorder; SP, Specific Phobia.

^a^ Revised Social Anxiety Scale for Children.

^b^ Revised Fear Survey Schedule for Children.

^c^ Children's Depression Inventory.

^d^ Child Behavior Checklist.

*p < .05, **p < .01, ***p < .001.

### Valence ratings

The ANOVA yielded a significant main effect of Group, *F*(2, 149) = 8.24, *p* < .001, $\eta_{p}^{2}$ = .100, and Context Valence, *F*(1.34, 199.53) = 539.36, *p* < .001, $\eta_{p}^{2}$ = .784 and a significant Age × Context Valence interaction, *F*(1.34, 199.53) = 4.69, *p* = .022, $\eta_{p}^{2}$ = .030; other effects: *F*s <3.08, *p*s > .08. Youth with SAD rated all faces more negatively compared to HCs, *t*(149) = −4.05, *p* < .001, and compared to youth with SP at trend level, *t*(149) = −2.24, *p =* .081. In addition, all groups rated positively contextualized faces as most positive, followed by faces with neutral, and then faces with negative context valence, *t*s(149) > 16.86, *p*s < .001. Post hoc analyses on the Age × Context Valence interaction showed that younger participants rated faces in negative contexts as more negative than older participants, *t*(149) = −2.67, *p* = .008. See Table 3 for mean ratings per Group and Context Valence.

**Table 3:** Mean subjective valence ratings per group for neutral faces in negative, neutral and positive contexts.

Context valence	SAD	SP	HC
	*M* (*SD*)	*M* (*SD*)	*M* (*SD*)
Negative	2.50 (1.09)	2.73 (1.69)	2.79 (1.38)
Neutral	5.14 (0.86)	5.50 (1.50)	5.94 (1.03)
Positive	6.50 (1.22)	6.93 (1.21)	7.34 (1.28)

HC, Healthy Control; SAD, Social Anxiety Disorder; SP, Specific Phobia.

### P100

Mean ERP amplitudes per Group, Age and Context Valence can be found in Table S1. For the P100, the ANOVA revealed significant main effects of Age, *F*(1, 149) = 30.49, *p* < .001, $\eta_{p}^{2}$ = .170 and Context Valence, *F*(1.98, 295.72) = 6.78, *p* = .007, $\eta_{p}^{2}$ = .044; other effects: *F*s < 1.32, *p*s > .44. Younger participants showed higher P100 amplitudes than older participants. Across groups, P100 amplitudes were enhanced for negatively contextualized faces compared to faces with neutral context information, *t*(149) = 3.72, *p* < .001, and positive context information at trend level, *t*(149) = 2.31, *p* = .058.

### N170

**Figure 3:**
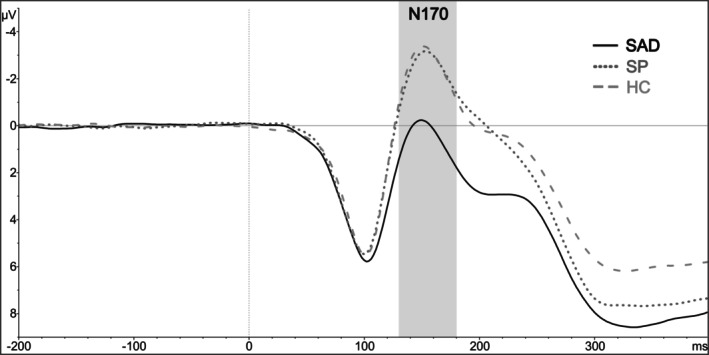
Illustrating the grand mean of the N170 in response to neutral faces averaged across trials with negative, neutral and positive context valence, as well as across P7 and P8 electrodes. HC, Healthy Control; SAD, Social Anxiety Disorder, SP, Specific Phobia

For the N170, there was a significant Group effect, *F*(2, 149) = 6.94, *p* = .007, $\eta_{p}^{2}$ = .085; other effects: *F*s < 5.05, *p*s > .094. Youth with SAD had lower N170 amplitudes than HCs, *t*(149) = 3.57, *p* = .002, and controls with SP, *t*(149) = 2.68, *p* = .022, with no difference between control groups, *t*(149) = 0.51, *p* = .87 (Figure 3).

### LPP

**Figure 4:**
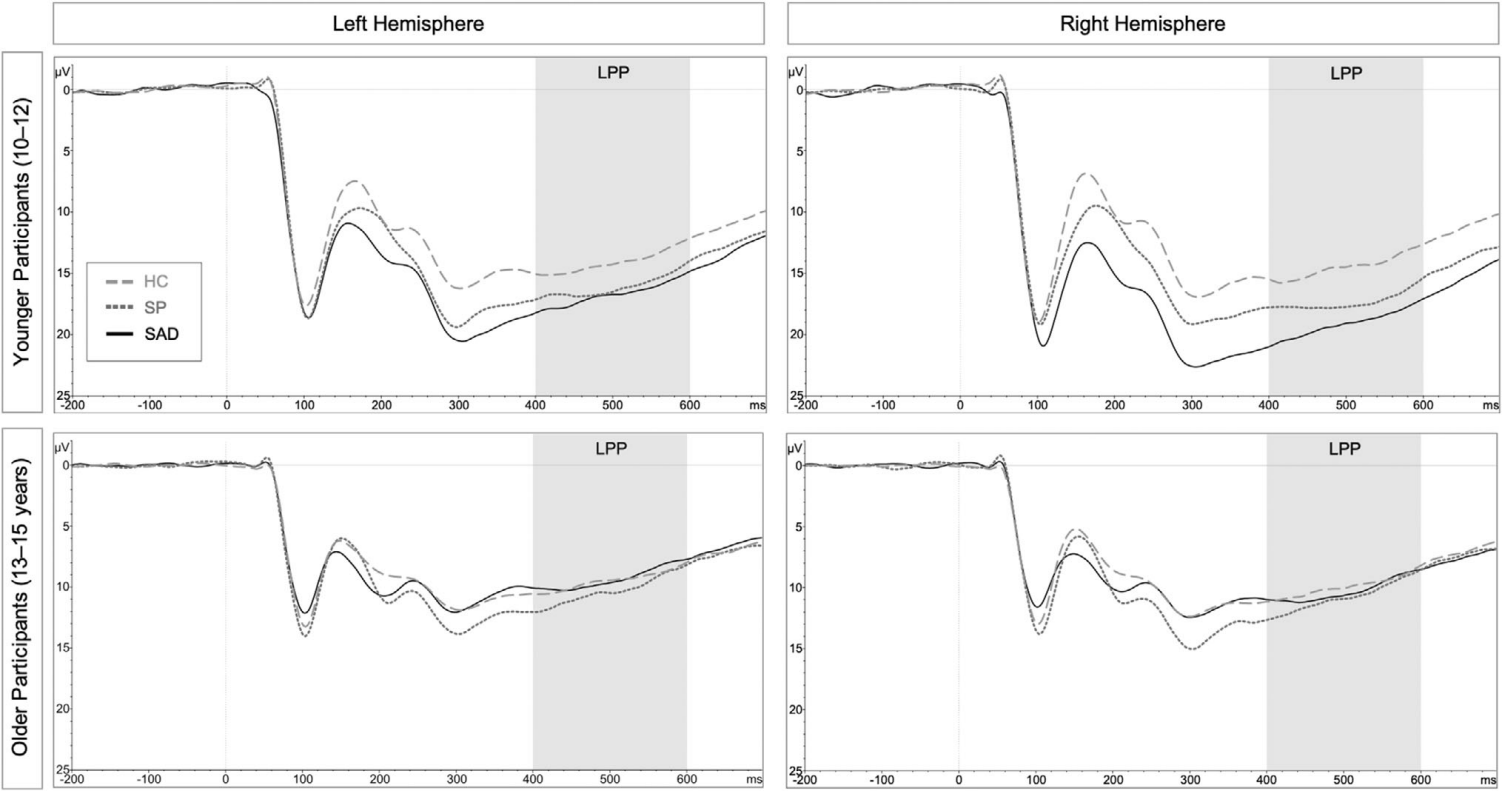
Depicting the interaction between Group, Age and Hemisphere on LPP amplitudes (averaged across Context Valence because the group difference between younger children with SAD and HCs in the right hemisphere was significant for negative, neutral and positive valences). Left hemisphere = O1 and PO3; right hemisphere = O2 and PO4. HC, Healthy Control; SAD, Social Anxiety Disorder; SP, Specific Phobia

The ANOVA revealed significant main effects of Age, *F*(1, 149) = 51.22, *p* < .001, $\eta_{p}^{2}$ = .256, Context Valence, *F*(1.97, 292.93) = 16.85, *p* < .001, $\eta_{p}^{2}$ = .102, and Hemisphere, *F*(1, 149) = 20.29, *p* < .001, $\eta_{p}^{2}$ = .120. Additionally, the 4‐way interaction between Group, Age, Context Valence and Hemisphere reached significance, *F*(3.95, 293.92) = 3.36, *p* = .045, $\eta_{p}^{2}$ = .043; other effects: *F*s < 3.20, *p*s > .14. Post hoc comparisons for the interaction showed that only younger children with SAD had higher LPPs than younger HCs in the right hemisphere in response to neutral faces with negative, neutral and positive context valence: *t*(149) = 2.68, *p* = .022, *t*(149) = 3.41, *p* = .002, and *t*(149) = 2.53, *p* = .034 (Figure 4). Other comparisons did not reach significance, *t*s(149) < 2.04, *p*s > .10, but there was a trend for younger children with SAD to also show higher LPPs than younger HCs in the left hemisphere in response to negatively contextualized faces, *t*(149) = 2.26, *p* = .065.

### Exploratory analyses: Subjective arousal, age effects and symptom severity

Mean subjective arousal ratings reflected the average of the subjective arousal levels rated before and after the EEG experiment (*n* = 154). They varied as a function of Group, *F*(2, 151) = 14.56, *p* < .001, $\eta_{p}^{2}$ = .162; youth with SAD reported higher arousal (*M* = 1.53, *SD* = 1.64), compared to youth with SP (*M* = 0.84, *SD* = 1.55) and HCs (*M* = 0.22, *SD* = 0.53), *t*s(151) > 2.56, *p*s < .031. Control groups differed at trend level, *t*(151) = 2.35, *p* = .052. Arousal ratings were not systematically correlated with ERPs (*p*s > .10).

To further investigate age as a moderator, we examined whether our age groups differed in their symptom severity. Age effects regarding psychometric measures were not significant in the SAD, SP or HC groups (*p*s > .11) but younger participants with SAD showed a trend towards higher self‐reported phobic fears (Mdn = 66) compared to older participants with SAD (Mdn = 52), *W* = 454, *p* = .059, and the same pattern emerged in participants with SP (younger: Mdn = 49; older: Mdn = 34.5, *t*(38.7) = 1.86, *p* = .070).

To follow‐up on the observed group differences, we examined potential associations between symptom severity, as indexed by psychometric measures and outcome measures with significant group effects (Table 4). For all participants, higher levels of social anxiety, phobic fears, depressive symptoms, general psychopathology and internalizing symptoms were associated with more negative valence ratings for faces and smaller N170 amplitudes, although with small effect sizes. Higher levels of phobic fears were associated with larger right hemisphere LPPs in younger participants.

**Table 4:** Correlations between psychometric measures and outcomes with a significant main or interaction effect of Group

	Valence rating	N170	LPP (younger age group, right hemisphere)
Social anxiety^a^	−.24***	.14**	.15
Phobic fears^b^	−.18**	.14**	.16*
Depressive symptoms^c^	−.13*	.18**	.08
General psychopathology^d^	−.20***	.13*	.10
Internalizing symptoms	−.23***	.17**	.06
Externalizing symptoms	−.0	.10	.13

Due to nonnormality and ranks, Kendall's tau was computed. For the N170, more positive values reflect smaller N170 amplitudes. For the LPP, group differences were only significant in younger participants (10–12 years) in the right hemisphere and thus correlations were calculated for this subsample (*n* = 79). Note that the German version of the questionnaires was used. LPP, late positive potential.

^a^ Revised Social Anxiety Scale for Children.

^b^ Revised Fear Survey Schedule for Children.

^c^ Children's Depression Inventory.

^d^ Child Behavior Checklist.

*p < .05, **p < .01, ***p < .001.

## Discussion

The aim of the present study was to investigate the influence of social context information on the subjective and neural processing of children's neutral faces in childhood SAD. Children and adolescents aged 10–15 years with SAD, clinical controls with SP, and HCs viewed neutral faces preceded by auditive contextual sentences with negative, neutral or positive valence. Overall, valence ratings and neural responses (P100 and LPP) to neutral faces were modulated by the valence of the context information, supporting the effects of our experimental manipulation and the importance of contextual cues in the processing of neutral faces.

Importantly, our results provide novel evidence that face processing biases can be found in youth with SAD, seemingly independent of the context valence. As hypothesized, participants with SAD rated negatively contextualized faces more negatively than HCs. Unexpectedly, they also rated neutral faces with positive and neutral context information more negatively. Contrary to our hypothesis, valence ratings did not differ between youth with SAD and clinical controls. Neurally, and in line with our hypothesis, youth with SAD showed reduced N170 amplitudes compared to both control groups, irrespective of the context valence. Finally, we expected increased LPPs for negatively contextualized faces in SAD. This was only partially confirmed: Only younger children with SAD (10–12 years) showed higher lateralized LPPs to all contextualized faces compared to younger HCs, with no differences compared to clinical controls.

Our study is the first known to show altered neural processing of neutral faces in SAD during childhood and adolescence, which seems to be independent of context valence. More specifically, youth with SAD had lower N170 amplitudes in response to neutral faces paired with negative, neutral or positive context information when compared to both clinical controls with SP and HCs. Our findings are consistent with those of Wieser and Moscovitch (2015), who used the same paradigm for socially anxious adults. Lower N170 amplitudes have also already been reported in a non‐clinical sample of socially anxious children (Wauthia et al., 2023) and in the same sample of youth with SAD but in response to emotional adult faces and only in comparison with HCs (Rauschenbach et al., 2024). Overall, lower N170 amplitudes have been interpreted as indicative of reduced structural face encoding or associated with the processing of more unrefined emotional cues necessary to quickly assess an emotional expression (Riwkes, Goldstein, & Gilboa‐Schechtman, 2015; Vuilleumier, Armony, Driver, & Dolan, 2003; Wieser & Moscovitch, 2015). The less detailed facial processing or cognitive avoidance observed in this instance may result in a more threatening perception due to the incomplete processing of the stimulus. In contrast to the N170, we did not find group differences regarding the P100. While this is inconsistent with findings from adult studies (Harrewijn et al., 2017), it is in line with results of previous studies on neural face processing in childhood SAD (Keil et al., 2018, 2022; Rauschenbach et al., 2024; Schwab & Schienle, 2017a, 2017b).

Regarding later processing stages, only younger children with SAD showed higher lateralized LPP amplitudes than younger HCs in response to all neutral faces, irrespective of the context valence. In line with these findings, several paediatric studies have also reported an association between SAD and higher LPPs in response to emotional faces, which was interpreted as an index of sustained cognitive processing (Kujawa et al., 2015; Schwab & Schienle, 2017a, 2017b). Interestingly, the modulation of the LPP as a function of clinical social anxiety has only been reported in rather young samples and similar age effects were observed in the same sample in response to emotional adult faces (Rauschenbach et al., 2024). In line with these findings, differences in LPP amplitudes between youth with SAD and HCs were not found in slightly older samples (Keil et al., 2018, 2022). Younger children generally show heightened electrocortical reactivity (e.g., Kujawa, Klein, & Hajcak, 2012), which may further be amplified by clinical social anxiety. Thus, childhood could potentially represent a sensitive time period for neural biases and the development of SAD. Consequently, the findings underline the need for developmental approaches and the investigation of age effects represents an important endeavour for future studies. As associations between ERPs and internalizing symptoms have already been found in children aged 5–10 years (Dennis & Hajcak, 2009), longitudinal studies including younger children with clinical and subclinical SAD may provide further insight into the early onset of social anxiety.

In the current sample, higher symptom severity regarding fear‐related measures, depressive symptoms, as well as internalizing symptoms and overall psychopathology, was associated with more negative valence ratings and smaller N170 amplitudes. Additionally, higher phobic fears were associated with larger lateralized LPPs in younger participants. These findings may indicate a small general effect of symptom severity and overall psychopathology. At the same time, youth with SP reported higher levels of phobic fears and overall psychopathology compared to HCs, but both control groups did not differ on any behavioral or neural outcome. While previous studies reported higher LPPs in children (Leutgeb, Schäfer, Köchel, Scharmüller, & Schienle, 2010) and adults (Michalowski et al., 2009) with spider phobia compared to HCs, these effects were limited to phobia‐relevant stimuli, and the studies did not include social stimuli, limiting conclusions about transdiagnostic face processing biases. To further investigate the influence of symptom severity, future research would benefit from the inclusion of a clinical control group with similar symptom severity and comorbidities to SAD (e.g., youth with generalized anxiety disorder), and paradigms should include disorder‐relevant stimuli for clinical controls.

Surprisingly, although our results support the idea that neutral face processing in childhood and adolescence may be context‐dependent, the influence of context information was not modulated by social anxiety. This was found for both subjective and neural responses. Several explanations are plausible.

First, in addition to the fear of negative evaluation, more recent approaches have emphasized fears related to positive evaluations and shown their distinct contribution to symptoms of social anxiety in children (Poole, Hassan, & Schmidt, 2022). Accordingly, youth with SAD in our sample may have reacted in a biased manner to all neutral faces since they were all embedded in an evaluative context, in which another child makes a statement pertaining to the participant, and therefore, all context phrases may have been perceived as threatening. Valence ratings support this hypothesis, as youth with SAD rated all neutral faces as more negative compared to HCs.

Second, previous studies suggest that youth show a negativity bias in their response to neutral faces, evident in their affective ratings, as well as physiological correlates, including the activity in corticolimbic emotion‐processing structures and corrugator muscle activity (Marusak, Zundel, Brown, Rabinak, & Thomason, 2017; Tottenham, Phuong, Flannery, Gabard‐Durnam, & Goff, 2013). Consequently, youth seem to perceive neutral faces as having negative affect; thus, contextual cues may be less crucial. Together with these previous findings, our results suggest that neutral faces are not processed as neutral, particularly in youth with SAD, and therefore, it may not be advisable to use them as a neutral baseline.

Limiting the generalizability of our results, we only presented static pictures and did not assess face processing in an ecologically valid manner (e.g., interactions, nonverbal social cues). Although subjective arousal ratings were generally low, youth with SAD reported higher levels than controls. Statistical analyses showed no association between subjective arousal ratings and ERPs, but we cannot rule out a possible effect on our results. Lastly, our sample size did not allow us to examine interactions between age and gender, which remain an important endeavour for future studies.

## Conclusion

Overall, our results provide further evidence that behavioural and neural face processing biases are already present in children and adolescents with SAD and may play a crucial role in the maintenance of the disorder. In addition, our findings underline the need for developmental approaches and show that neutral child faces are not processed as neutral, especially in younger children with SAD.

## Ethical information

All parents provided informed consent, and assent was obtained from participating youth. The project was approved by the ethics committee of Leipzig University (approval number: 037/19‐ek; date of approval: 16.02.2019).

**Key points**

**What's known**

- Face processing biases and the fear of evaluation play a key role in social anxiety disorder (SAD).

**What's new**

- In a large sample of youth (aged 10–15), those with SAD had lower N170 amplitudes compared to clinical and healthy controls in response to neutral faces independent of the context valence.
- Younger children with SAD also showed increased LPP amplitudes compared to healthy controls – suggesting age‐related differences in neural processing.

**What's relevant**

- While youth with SAD already show subjective and neural processing biases in response to neutral faces, the effect of context information seems to be independent of psychopathology. The findings underline the need for developmental approaches.

## Supporting information


**Table S1.** Group means and SDs in μV for all ERP components separately for each Age group and Context Valence averaged across ERP‐specific electrodes.

## Data Availability

The data that support the findings of this study are available from the corresponding author upon reasonable request.
