# Supplementary material for: Effects of social context information on neural face processing in youth with social anxiety disorder
Source: J Child Psychol Psychiatry. 2025 Aug 4;67(5):620–30. doi: 10.1111/jcpp.70026 (PMC13102049; doi:10.1111/jcpp.70026)
Supplement: Supplementary file 1 — Table S1. Group means and SDs in μV for all ERP components separately for each Age group and Context Valence averaged across ERP‐specific electrodes. [file JCPP-67-620-s001.docx]

**Supporting Information**

**Table S1**

Group means and SDs in μV for all ERP components separately for each Age group and Context Valence averaged across ERP-specific electrodes.

|  |  |  | SAD | | SP | | HC | |
| --- | --- | --- | --- | --- | --- | --- | --- | --- |
|  |  |  | *M (SD)* | | *M (SD)* | | *M (SD)* | |
| P100 | Younger | |  | |  | |  | |
|  |  | Negative | 17.93 (7.40) | 16.24 (5.01) | | 16.91 (7.02) | |  |
|  |  | Neutral | 17.12 (7.65) | 16.02 (5.27) | | 15.23 (7.39) | |  |
|  |  | Positive | 17.33 (7.00) | 16.06 (3.91) | | 16.29 (6.99) | |  |
|  | Older |  |  |  | |  | |  |
|  |  | Negative | 10.52 (5.11) | 13.33 (2.78) | | 11.63 (4.35) | |  |
|  |  | Neutral | 10.36 (5.45) | 12.59 (2.64) | | 11.23 (4.53) | |  |
|  |  | Positive | 10.58 (4.83) | 12.49 (3.36) | | 11.34 (4.76) | |  |
| N170 | Younger | |  |  | |  | |  |
|  |  | Negative | 0.60 (5.76) | -3.47 (4.99) | | -3.34 (4.49) | |  |
|  |  | Neutral | 0.40 (5.17) | -3.46 (4.58) | | -3.73 (4.85) | |  |
|  |  | Positive | 0.32 (5.30) | -3.37 (5.33) | | -3.95 (4.40) | |  |
|  | Older |  |  |  | |  | |  |
|  |  | Negative | 0.70 (4.27) | -0.46 (4.11) | | -1.36 (4.37) | |  |
|  |  | Neutral | 0.38 (3.79) | -0.82 (3.80) | | -1.51 (4.33) | |  |
|  |  | Positive | 0.43 (4.44) | -0.91 (4.01) | | -1.41 (4.31) | |  |
| LPP | Younger | |  |  | |  | |  |
|  |  | Negative | 18.98 (6.88) | 17.63 (6.47) | | 14.96 (6.90) | |  |
|  |  | Neutral | 17.25 (6.17) | 16.10 (5.93) | | 13.40 (6.63) | |  |
|  |  | Positive | 17.64 (5.59) | 16.57 (6.17) | | 14.56 (7.21) | |  |
|  | Older |  |  |  | |  | |  |
|  |  | Negative | 10.00 (4.17) | 11.23 (4.62) | | 10.03 (4.57) | |  |
|  |  | Neutral | 9.52 (3.90) | 9.98 (4.35) | | 9.25 (4.36) | |  |
|  |  | Positive | 9.98 (3.84) | 10.53 (4.56) | | 9.93 (4.70) | |  |

*Note.* SAD = Social Anxiety Disorder, SP = Specific Phobia, HC = Healthy Controls, LPP = Late Positive Potential, younger age group = aged 10-12 years, older age group = aged 13-15 years.
